# Common Minor Histocompatibility Antigen Discovery Based upon Patient Clinical Outcomes and Genomic Data

**DOI:** 10.1371/journal.pone.0023217

**Published:** 2011-08-09

**Authors:** Paul M. Armistead, Shoudan Liang, Hua Li, Sijie Lu, Cornelis A. M. Van Bergen, Gheath Alatrash, Lisa St. John, Sally A. Hunsucker, Stefanie Sarantopoulos, J. H. Frederik Falkenburg, Jeffrey J. Molldrem

**Affiliations:** 1 Section of Transplantation Immunology, Department of Stem Cell Transplant and Cellular Therapy, M.D. Anderson Cancer Center, Houston, Texas, United States of America; 2 Department of Bioinformatics and Computational Biology, M.D. Anderson Cancer Center, Houston, Texas, United States of America; 3 Laboratory of Experimental Hematology, Leiden University Medical Center, Leiden, The Netherlands; 4 Lineberger Comprehensive Cancer Center, University of North Carolina, Chapel Hill, North Carolina, United States of America; Rega Institute, University of Leuven, Belgium

## Abstract

**Background:**

Minor histocompatibility antigens (mHA) mediate much of the graft vs. leukemia (GvL) effect and graft vs. host disease (GvHD) in patients who undergo allogeneic stem cell transplantation (SCT) [1], [2], [3], [4]. Therapeutic decision making and treatments [5] based upon mHAs will require the evaluation of multiple candidate mHAs and the selection of those with the potential to have the greatest impact on clinical outcomes. We hypothesized that common, immunodominant mHAs, which are presented by HLA-A, B, and C molecules, can mediate clinically significant GvL and/or GvHD, and that these mHAs can be identified through association of genomic data with clinical outcomes.

**Methodology/Principal Findings:**

Because most mHAs result from donor/recipient cSNP disparities, we genotyped 57 myeloid leukemia patients and their donors at 13,917 cSNPs [6]. We correlated the frequency of genetically predicted mHA disparities with clinical evidence of an immune response and then computationally screened all peptides mapping to the highly associated cSNPs for their ability to bind to HLA molecules. As proof-of-concept, we analyzed one predicted antigen, T4A, whose mHA mismatch trended towards improved overall and disease free survival in our cohort. T4A mHA mismatches occurred at the maximum theoretical frequency for any given SCT. T4A-specific CD8+ T lymphocytes (CTLs) were detected in 3 of 4 evaluable post-transplant patients predicted to have a T4A mismatch.

**Conclusions/Significance:**

Our method is the first to combine clinical outcomes data with genomics and bioinformatics methods to predict and confirm a mHA. Refinement of this method should enable the discovery of clinically relevant mHAs in the majority of transplant patients and possibly lead to novel immunotherapeutics [5].

## Introduction

Allogeneic stem cell transplant (SCT) is curative for many patients with advanced hematologic malignancies, and in recent years, the role of donor-derived immunity (i.e. GvL) has been shown to be crucial in maintaining durable remissions [7], [8], [9], [10]. One strong piece of evidence to support the role of donor-derived immunity in the setting of SCT is the 20-year old observation that syngeneic SCT is associated with 3-fold higher rates of relapse when compared to HLA-matched allogeneic SCT [2].

The fact that syngeneic SCT has a higher relapse rate compared to HLA-matched, allogeneic SCT [2], and that this increased relapse rate rivals that of HLA-matched allogeneic SCT with donor T cell depletion [4], is indirect evidence that much of the clinically observed GvL effect is mediated by donor T cell reactivity against patient mHAs (presented on HLA-A, B, C molecules) that differ in peptide sequence from the homologous mHAs present in the donor [1], [6]. These peptide differences between donor and patient that may account for the curative GvL are often the result of non-synonymous coding single nucleotide polymorphisms (cSNPs) that yield a protein with a single amino acid difference between the donor and patient [1], [6]. Donor T cells obtained from a donor who is homozygous for a certain allele in a given HLA epitope would be predicted to be intolerant towards an HLA epitope derived from the alternate allele. In such cases, donor T cells would be predicted to mount an immune response upon exposure to the alternate allele epitope if it is expressed by the patient. This mechanism of allo-immunity has been confirmed in several well-characterized mHAs such as HA-1 and LB-ADIR-1F [3], [11], [12].

Traditional mHA discovery methods begin with the establishment of a donor-derived T cell clone obtained from the patient post-SCT that reacts to an unknown antigen expressed on an antigen presenting cell usually, but not always, obtained from the recipient [5], [13], [14], [15], [16], [17]. These cloning approaches, by themselves, are unable to provide immediate information regarding the minor antigen's HLA-restriction, chemical structure, tissue restriction or allele frequency – characteristics that are important considerations if mHA therapeutics are to be developed clinically. Determination of these properties requires further experimentation that can only be performed after the initial cloning [12], [18]. Because each SCT has thousands of potential mHA mismatches: some unique to that particular donor patient pair and others common in multiple transplant pairs, each with varying levels of immunogenicity, the individual characterization of T cell clones is insufficient to screen all of the potential mHA in the human population. If therapeutic decision-making that is based on minor histocompatibility antigen analysis is to become part of standard clinical practice, methods must be developed to efficiently evaluate multiple candidate mHAs and determine which ones have the properties (e.g. antigen frequency, tissue expression, association with clinical outcomes) most likely to provide the greatest impact on clinical outcomes for large patient cohorts [3], [19], [20]. With the advent of robust high-throughput genomic platforms, cSNP analysis of patient populations is being performed in many diverse fields, and should be applicable to mHA discovery [14], [15], [21]. Given the large size and genetic diversity throughout the human population, the number of allelic mismatches occurring between any given HLA-identical donor/patient pair is prohibitively large to perform a detailed characterization of each potential mHA mismatch and its resulting capacity to generate an immune response [22]; however, we hypothesized that common, immunodominant mHAs that mediate clinically significant effects can be discovered by analyzing clinical outcomes from a cohort of SCT patients, correlating the clinical outcomes with genetically predicted mHA mismatches, and using bioinformatics platforms to simplify the identification of the actual HLA epitopes.

To test our hypothesis, we applied this general approach to a SCT patient cohort with the aim of identifying a common, immunodominant mHA. In this pilot study, we evaluated 13,917 cSNPs and measured the association between donor/recipient cSNP disparities and recipient outcomes. In a cohort of 57 myeloid leukemia patients, we identified a new common mHA, T4A, which was associated with a trend toward improved OS, DFS, and relapse rate. T4A immunogenicity was confirmed by identifying functional CD8+ T cells in post-SCT patients. Additionally, T4A possesses several properties that make it a good target mHA: 1) T4A mHA mismatch occurs at the theoretical maximum frequency for any given SCT population and 2) T4A expression is highly restricted to hematopoietic tissue and leukemia.

## Materials and Methods

### Patient Cohort Assembly

Written informed consent for blood sample collection was obtained from all patients and donors through an M.D. Anderson IRB-approved protocol (LAB99-062). Research was conducted according to the principles in the Declaration of Helsinki. We initially attempted to create a large patient cohort consisting of HLA-A2+ patients with myeloid leukemia (AML, CML, MDS) who had undergone an HLA-matched related HSCT and had not developed a GvHD beyond either acute GvHD (aGvHD) grade 1 or limited chronic GvHD (cGvHD). We were unable to obtain a sufficiently large number of samples using these criteria so we decided to add myeloid leukemia patients who had developed either aGvHD > grade 1 or extensive cGvHD. The addition of these patients increased our test cohort from 36 to 57 patients. We obtained paired pre-transplant donor and patient peripheral blood mononuclear cells (PBMC) from the tissue bank of the Department of Stem Cell Transplantation and Cellular Therapy under an IRB approved protocol. For 9 of the 57 samples we used post-transplant, 100% donor chimeric PBMC samples as the “donor sample”. We extracted genomic DNA from the frozen PBMC samples using QIAamp (Qiagen, Valencia, CA) Blood DNA extraction kits according to the manufacturer's instructions.

### Donor/Patient Genotyping

Genomic DNA from the 57 patients and their matched donors was analyzed at 13,917 well-annotated cSNPs using Illumina NS-12 microarrays. This analysis was performed at the University of Texas at Houston School of Medicine microarray core facility.

### Peptide synthesis

Candidate peptides were synthesized by the M.D. Anderson peptide core facility using traditional f-moc chemistry, and peptides for the iTopia Epitope Discovery System were synthesized by Bioynthesis (Lewisville, TX). All peptides were purified by HPLC, and molecular weights were confirmed by mass spectrometry.

### Epitope binding assays

Peptides were dissolved in DMSO and diluted with milli-Q water to reach a stock concentration of 1 mM peptide with 1% DMSO. TAP deficient T2 cells were incubated in complete media (CM) consisting of RPMI supplemented with 10% FBS and 1% penicillin/streptomycin (P/S) at a concentration of 1×10^6^ cells/mL and a viability of >90%. The cells were then washed 3 times in serum-free RPMI with 1% P/S and resuspended at a concentration of 1×10^6^ cell/mL. Candidate epitope peptides were added to make a final concentration of 100 µM peptide. The T2 cells were incubated for 18 h in 5% CO_2_ at 37 °C. After incubation, T2 cells were washed in sterile phosphate buffered saline (PBS) twice and incubated with unconjugated BB7.2 antibody (10 µL purified hybridoma supernatant) for 30 min at 4°C. The cells were washed with sterile PBS once and then incubated for 15 min at 4°C with 5 µL FITC-labeled goat anti-mouse IgG antibody (Caltag, Burlingame, CA). Mean fluorescence intensity in the FITC channel was measured for live T2 cells in all samples. All assays were performed with a no-peptide control and a µM PR1 (VLQELNVTV) peptide loaded positive control.

Additional characterization of the P12 peptide (renamed T4A) binding to HLA-A0201 was performed using the iTopia Epitope Discovery System (Beckman Coulter, Miami, FL), following the manufacturer's protocol. To determine the ability of the T4A and T4E (the alternate allele) peptides to bind HLA-A0201, peptides were incubated in duplicate in HLA-A0201 coated wells at a concentration of 11 µM at 21°C overnight, and% peptide binding was calculated relative to the positive control peptide (FLPSDFFPSV). Per the manufacturer, peptides binding ≥30% of the positive control are candidate HLA-A0201 epitopes. To measure T4A binding affinity, the T4A peptide and control peptide were incubated in separate HLA-coated wells at peptide concentrations ranging from 10^−4^ to 10^−8^ M at 21°C overnight. Results from duplicate wells were graphed relative to the positive control peptide, and the ED_50_ was determined using GraphPad Prism's nonlinear regression, ‘log (agonist) versus response –variable slope (four parameter)’ curve. For off-rate analysis, T4A peptide and the control peptide were incubated in separate HLA-coated wells at a concentration of 11 µM at 21°C overnight, then washed and incubated at 37°C and read over the course of 8 hrs. Results from duplicate wells were graphed relative to the positive control peptide as 100% binding at each time point. The t_1/2_ was calculated using GraphPad Prism's nonlinear regression, ‘dissociation – one phase exponential decay’ curve.

### Tetramer Synthesis and Flow Cytometry

Tetramers were made, in our laboratory, for all of the peptides that bound HLA-A2 using the T2 binding assay according to published protocols [23]. Briefly, peptide loaded monomers were made in a 100 mL reaction consisting of 1 mg HLA-A0201 monomer containing a biotinylation recognition site, β2-microglobulin, and 1–2 mg of the peptide. After 3 days of incubation at 4 °C, the monomers were purified by size-exclusion chromatography on a Pharmacia FPLC and subsequently biotinylated using BirA ligase. The biotinylated monomers were purified by ion-exchange chromatography, and tested for purity by SDS-PAGE. Monomers were then reacted with phycoerythrin (PE) conjugated streptavidin and subsequently purified on 100 kDa membranes to yield PE-conjugated tetramers.

Frozen post-transplant PBMC patient samples were thawed and washed once in CM and once in PBS. Cell counting and viability was performed, and approximately 1×10^6^ cells (when possible) were incubated with 2 µL each of PE-Cy5-conjugated CD4, CD14, CD16, CD19 antibodies (‘dump’ channel), 1 µL FITC-conjugated CD8 antibody (all antibodies Caltag, Burlingame, CA), and 2.5 µg PE-conjugated tetramer at 4°C for 30 mins. Two µL aqua live/dead stain (Invitrogen, Carlsbad, CA) were added 25 mins into the labeling procedure. Single stain controls were used for compensation purposes. Samples were washed once after incubation and analyzed immediately on a Cyan flow cytometer. Tetramer+ cells were enumerated in the live (aqua-), lymphocyte (FS/SS), CD8+, ‘dump’− gate.

### Cytokine secretion assay

Normal donor buffy coats that served as negative controls were purified by ficol-hypaque purification and tested for HLA-A2 expression using BB7.2 antibody with a FITC-labeled antimouse IgG secondary antibody. Each HLA-A2+ buffy coat was genotyped at the rs9876490 (ref. seq. for the T4A peptide) SNP. 1×10^6^ PBMC from an HLA-A2+ buffy coat containing an AC genotype (predicted to be tolerant to the T4A peptide) and a ficol-hypaque sample obtained from the T4A gIR+ post-SCT evaluated in Figure 3C were incubated for 6h with 1×10^6^ T4A pulsed T2 cells, unpulsed T2 cells, or 2 µL OKT3 antibody. Each sample was incubated in 1 well of a 96 well plate containing 200 µL of CM. After 6 h supernatant was removed, and submitted for Luminex analysis measuring IFN-γ and TNF-α secretion.

### Tetramer-Cytokine Flow Cytometry

B lymphocytes from healthy donors expressing HLA-A0201 were immortalized using Epstein-Barr virus infection to generate EBV-LCLs. TRIM42 protein expression in each LCL was confirmed by western blot. Genomic DNA was isolated from each EBV-LCL, and PCR was used to amplify DNA flanking the T4A associated cSNP (rs9876490). Conventional Sanger sequencing was performed to determine the genotype at rs9876490 for each EBV-LCL. One EBV-LCL line had a genotype predicted to produce T4A peptide (T4A+), and another EBV-LCL had a genotype predicted to only produce the alternate peptide (T4A−).

An HLA-A2/T4A-APC tetramer was synthesized in our laboratory as described above. Frozen PBMC samples from post-SCT, T4A gIR+, patients were thawed, and 2.5 µg of tetramer were added to 1×10^6^ PBMC and incubated for 30 min on ice in the dark. The PBMC were washed in PBS and resuspended in CM. Tetramer stained PBMC were incubated with 1×10^6^ T4A+ or T4A- EBV-LCLs in a 1∶1 ratio in a well of a v-bottom 96-well plate. To each well was added 2 µL pure CD28 and 0.5 µL pure CD49d. The PBMC/EBV-LCL cultures were incubated for 1 hr at 37°C in 5% CO_2_, and then brefeldin A was added. The cells were incubated for an additional 5 hrs and then transferred to 4°C overnight. Media was removed and the cells were incubated in PBS +0.02% EDTA at 37°C for 10 min, then washed in PBS. Cells were stained with Live/Dead Fixable Aqua (Invitrogen, Carlsbad, CA) for 20 min on ice. Cells were then washed with PBS and resuspended in FACS Lyse (Beckton Dickinson, Franklin Lakes, NJ) solution for 10 mins at room temperature. Following this, the cells were again washed and resuspended in FACS PermII (Beckton Dickinson) solution for 10 mins at room temperature. The cells were washed once in PBS +1% BSA +0.02% sodium azide. The following fluorescently conjugated antibodies were added to each sample: 4 µL CD8-APC-H7 (Beckton Dickinson); 2 µL each CD4, CD14, CD16-Pacific Blue (Beckton Dickinson), CD19-Pacific Blue (Biolegend, San Diego, CA); 4 µL IFN-γ-PE-Cy7 (Becton Dickinson); 1 µL Aqua Live/Dead (Invitrogen). The cells were incubated for 30 mins at 4°C, and were then washed once in PBS +1% BSA +0.02% sodium azide. The cells were fixed in 200 µL of 1% paraformaldehyde and analyzed using a BD LSRII Fortessa flow-cytometer. Tetramer+, IFN-γ+ cells were enumerated in the live (aqua-), lymphocyte (FS/SS), CD8+, ‘dump’− gate.

### Survival Analysis

OS, DFS and relapse rates for the T4A gIR+ and T4A gIR- groups in the 57 patient cohort were analyzed using Kaplan-Meier survival curves [24]. Survival differences were compared using the log-rank test.

### Western Blot Analysis

Normal human tissue lysates (heart, testis, colon, skin, liver) prepared using RIPA buffer and SDS sample buffer were purchased from Prosci (Poway, CA). Four human AML samples, a PBMC sample and a Jurkat cell lysate were also prepared using RIPA buffer (50 mM Tris, pH 7.4, 150 mM NaCl, 1% sodium deoxycholate, 1 mM EDTA, 0.1% SDS, 1% Triton-X and Sigmafast Protease Inhibitor (Sigma-Aldrich, St. Louis, MO). Twenty µg of each lysate (Micro BCA protein assay, Thermo Scientific, Rockford, IL) were incubated with loading dye (2% SDS, 10% glycerol, 62.5 mM Tris, pH 6.8, 5% beta-mercaptoethanol and 0.002% bromophenol blue) in a total volume of 15 µL for 5 min at 98°C. Each sample was loaded onto a 4–12% NuPAGE gradient gel (Invitrogen, Carlsbad, CA) and electrophoresed at 100 mV for 140 min. The gel was then transferred to a PVDF membrane using a Mini-PROTEAN Trans-Blot Module (Biorad, Hercules, CA) at 250 mA for 70 min and blocked with 5% milk for 1 hr prior to incubation with the anti-TRIM42 antibody (Abcam, Cambridge, MA) at 1∶1000 dilution overnight at 4°C. The next day, the membrane was washed for 30 min in TBS-0.1% Tween and then incubated with horse-radish peroxidase conjugated anti-rabbit IgG antibody for 1 hr and developed using the Amersham ECL Western Blotting Detection kit (GE Healthcare, Piscataway, NJ). The membrane was reprobed with monoclonal anti-GADPH antibody (Sigma-Aldrich, St. Louis, MO).

Subcellular fractionation of AML blasts and normal granulocytes was performed using Proteoextract subcellular fractionation kit (Calbiochem, San Diego, CA). Five µg of each fraction (Bradford assay) were incubated with 6 µL beta-mercaptoethanol and 50 mM DTT in a total volume of 20 µL for 10 min at 100 °C. Each sample was loaded onto a 5–15% SDS-PAGE gradient gel (Biorad, Hercules, CA) and electrophoresed at 100 mV for 90 min. The gel was then transferred to a PME membrane using an electric gel blotter at 15 mV for 50 min and blocked with 2.5% milk for 1 hr. The membranes we then developed as described above.

## Results

### Patient characteristics

Table 1 shows the clinical characteristics of the 57 patient cohort. Following preliminary selection based upon database records of HLAA2+ type, disease, remission status and GvHD status we performed a more thorough chart review of all of the patients. From this review we found that 2 patients who had been classified as 10/10 HLA-match actually underwent HSCT from a related, but 1-antigen mismatch donor. Neither of these mismatches occurred in the HLA-A locus. The rates of acute and chronic GvHD in the cohort were lower than would be expected in a typical transplant cohort [25]; however, the lower GvHD incidence is a result of our initial attempts to capture only myeloid HLA-A2+ patients who did not have GvHD.

**Table 1:** Patient cohort characteristics.

Characteristics		Patients, N = 57 (%)
Median Age, years (range)		48 (21–66)
Gender		
	Male patient	37 (65)
		SMM = 20 (54)
	Female patient	20 (35)
		SMM = 9 (45)
Disease (%)		
	AML	29 (51)
		CR = 14 (48)
	CML	17 (30)
		CP = 12 (71)
	MDS	11 (19)
		CR = 0 (0)
Cytogenetics		
	Favorable	16 (28)
	Not favorable	41 (72)
Conditioning		
	Myeloablative	44 (77)
	Reduced intensity	13 (23)
Donor source		
	Bone Marrow	9 (16)
	Peripheral blood stem cell	48 (84)
Donor type		
	HLA-matched	55 (96)
	1 antigen mis-matched	2 (4)
Patient or Donor CMV+		49 (86)
GvHD Incidence		
	Acute ≥ grade 2	14 (25)
	Extensive chronic	11 (19)

SMM denotes a sex mismatched transplant.

CR denotes complete remission at the time of transplant.

CP denotes chronic phase at the time of transplant.

### Association of cSNP mHA disparities with clinical outcomes

**Figure 1:**
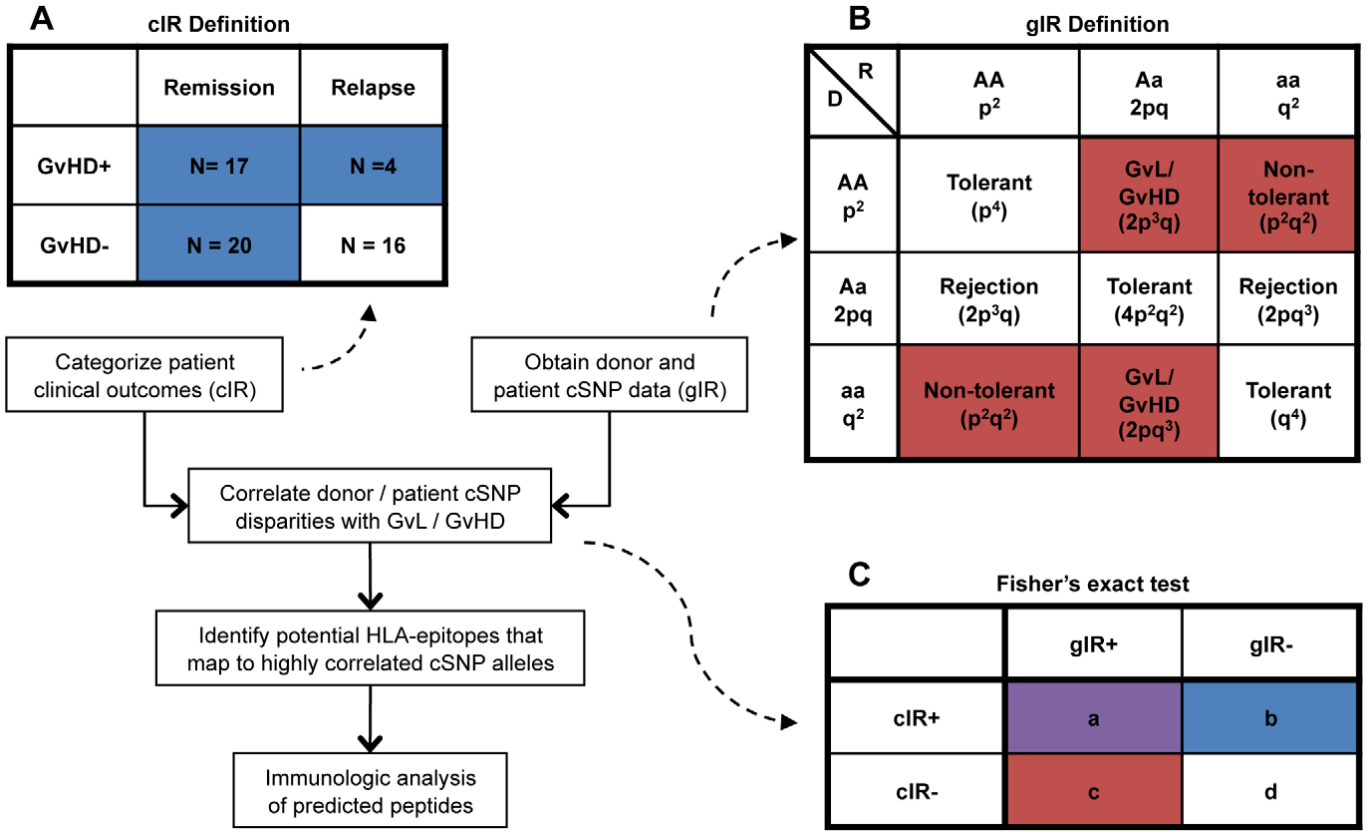
Schematic of mHA selection method. All 57 patients were classified by GvHD and remission status (1A). The patients in the remission, GvHD+; remission, GvHD-; and relapse, GvHD+ groups were considered as the clinical immune response cohort (cIR+, blue shaded boxes), and the relapse, GvHD- group was considered the clinical non-immune responder group (cIR-, unshaded box). All potential allele combinations for any cSNP in the setting of allogeneic SCT can be represented by a 3×3 table( 1B). Given a major allele ‘A’ and minor allele ‘a’ at any particular cSNP locus, then a donor (D) may be homozygous for the major allele (AA, genotype frequency  =  p^2^), heterozygous (Aa, genotype frequency  = 2pq), or homozygous for the minor allele (aa, genotype frequency  =  q^2^). The same assignments can be made for the recipient (R). Assuming potential minor histocompatibility antigens arise from non-synonymous cSNPs, then the genotype frequencies can be multiplied to give the predicted frequencies of a donor/recipient immune reaction against peptide antigens encoded by that locus. For a donor that is homozygous at a given cSNP, 3 potential immune responses can be predicted based on the genotype of the recipient: 1) tolerance, 2) non-tolerance in the GvL/GvHD direction, and 3) non-tolerance in both the GvL/GvHD and rejection directions. Scenarios 2 and 3 are defined as genetically predictive of an immune response (gIR+, red shaded boxes). No gIR is predicted if a donor is heterozygous at a given locus or if the donor and recipient share the same genotype (gIR-, unshaded boxes). The association between gIR+ and cIR+ for each allele in each cSNP was determined using Fisher's exact test (1C).

Figure 1 shows a schematic of how candidate cSNP alleles were identified as potential mediators of clinically significant immune responses. The 57 patients were clinically classified according to remission status and GvHD as described in Materials and Methods and represented in Figure 1A. The 4 subgroups can be considered to represent 2 broad clinical outcomes patients with evidence of a clinical immune response (cIR+, blue boxes), and patients without evidence of a clinical immune response (cIR-, unshaded box). Genomic DNA from each patient and his/her donor was genotyped at 13,917 cSNPs using Illumina NS-12 microarrays, and disparities between the donor and recipient pair were evaluated as illustrated in Figure 1B. For each cSNP a disparity involving a homozygous donor and genetically nonidentical (i.e. heterozygous at that cSNP or homozygous for the alternate allele) recipient was scored as genetically predictive of an immune response (gIR+, red boxes). All other genetic combinations were scored as genetically predictive of no immune response (gIR-, unshaded boxes). Each cSNP allele was evaluated by scoring the number of gIR+ and gIR- pairs in the entire patient cohort. Fisher's exact test was then applied for each cSNP to measure the association of gIR+ with cIR+ (Figure 1C). A strong association (i.e. low P-value) would be expected to occur if a cSNP allele yielded a large number of gIR+ disparities, and the gIR+ disparities preferentially occurred in the cIR+ group (Figure 1C), purple box. From the 57 patients in our test cohort we measured the association of cSNP gIR with cIR for 27,834 (13,917 cSNPs ×2 alleles/cSNP) alleles. The resultant list was ranked by P-value with the allele having the strongest association (rs2273959_T) between gIR and cIR having a P-value of 0.019 (Table 2). The 40 cSNPs that yielded the lowest P-value were further characterized in this pilot study.

**Table 2:** Peptides selected for evaluation.

Name	Recipient Allele	P-value	Gene	Peptide	IEDB	SY.	Donor allele	Donor peptide
*P1	rs2273959_T	0.019	SDCBP2	ASGDKIV**M**VV	642	17	rs2273959_C	ASGDKIV**V**VV
P2	rs2273959_C	0.019	SDCBP2	ASGDKIV**V**VV	1458	19	rs2273959_T	ASGDKIV**M**VV
*P3	rs4984906_A	0.021	WDR90	FLWDVLA**T**T	639	25	rs4984906_A	FLWDVLA**P**T
*P4	rs4479748_C	0.023	ENPP6	CMLKGRA**G**T	3303	19	rs4479748_C	CMLKGRA**S**T
P5	rs1801262_T	0.024	NEUROD1	**T**MNAEEDSL	860	21	rs1801262_T	**A**MNAEEDSL
P6	rs1864346_T	0.024	OR6N1	QV**T**EFIILG	2159	10	rs1864346_T	QV**A**EFIILG
P7	rs1864346_T	0.024	OR6N1	SQV**T**EFIILG	718	6	rs1864346_T	SQV**A**EFIILG
P8	rs1551122_A	0.025	C12orf64	IAMFAN**N**WSV	49	18	rs1551122_A	IAMFAN**S**WSV
P9	rs1551122_A	0.025	C12orf64	AMFAN**N**WSV	394	23	rs1551122_A	AMFAN**S**WSV
*P10	rs2303771_A	0.042	KLHDC4	LYNELYVYN**I**	2458	18	rs2303771_A	LYNELYVYN**T**
P11	rs2303771_A	0.042	KLHDC4	N**I**RKDTWTKV	847	20	rs2303771_A	N**T**RKDTWTKV
P12	rs9876490_C	0.043	TRIM42	GLYTYWSAG**A**	226	19	rs9876490_C	GLYTYWSAG**E**
P13	rs3748816_G	0.043	MMEL1	YILEE**T**NRRL	248	24	rs3748816_G	YILEE**M**NRRL
P14	rs3748816_G	0.043	MMEL1	ILEE**T**NRRL	2792	23	rs3748816_G	ILEE**M**NRRL
P15	rs9373475_T	0.044	FBOXO30	DLGD**M**KNDV	1308	22	rs9373475_T	DLGD**V**KNDV
P16	rs11649804_A	0.045	RAI1	V**T**FRTHSLHV	508	17	rs11649804_A	V**P**FRTHSLHV
P17	rs11649804_A	0.045	RAI1	QV**T**FRTHSL	2248	17	rs11649804_A	QV**P**FRTHSL
P18	rs214976_A	0.046	SYNE1	FLAS**V**EECRT	333	17	rs214976_A	FLAS**V**EECRT
P19	rs214976_A	0.046	SYNE1	S**V**EECRTEL	8780	19	rs214976_A	S**A**EECRTEL
P20	rs214976_A	0.046	SYNE1	S**V**EECRTELD	52688	7	rs214976_A	S**A**EECRTELD
*P21	rs1049534_T	0.046	THOC5	SIPP**I**FQLCL	3985	23	rs1049534_T	SIPP**V**FQLCL
P22	rs11567842_G	0.047	SLC13A2	IIGVLI**V**ALA	1494	20	rs11567842_G	IIGVLI**I**ALA
P23	rs11567842_G	0.047	SLC13A2	IIGVLI**V**AL	556	28	rs11567842_G	IIGVLI**I**AL

*Denotes peptides that could not be synthesized at high purity.

IEDB  =  IEDB SMM predicted IC_50_ (nM).

SY.  =  SYFPEITHI algorithm binding score.

The cSNP alleles with the strongest association between gIR+ and cIR+ in the 57-patient cohort are shown. For each cSNP, gIR analysis was performed twice, once with the major allele considered the recipient allele (i.e. the allele that encodes the peptide presented by the recipient that is recognized by the donor T lymphocyte repertoire) and once with the minor allele considered the recipient allele. The cSNPs are ranked by decreasing statistical association of gIR+ and cIR+ for the recipient allele (column 2). The peptide containing the recipient allele-associated amino acid (shown in bold) that was predicted to bind most tightly to HLA-A0201 according the IEDB (SMM) and SYFPEITHI algorithms are shown in column 5 with IEDB and SYFPEITHI prediction scores in columns 6 and 7 respectively. The corresponding donor allele and donor peptide (i.e. the peptide associated with the donor's homozygous allele are shown in columns 8 and 9 respectively.

### Bioinformatic methods accelerate candidate epitope discovery

Because of the large number of cSNPs tested the problem of false-positive results from multiple testing is significant in our method. To address this concern, we evaluated the 40 cSNPs with gIR+ most strongly associated with a cIR+ as possible mHAs by first using the Ensembl (www.ensembl.org) database, to map the cSNPs to a known peptide sequence (19 amino acids in length with the cSNP associated amino acid at position 10) that contained allelic variations resulting in an amino acid change. From the 40 cSNPs 16 were removed at this step because they could not be mapped to a known protein sequence with the given allelic variation. For the remaining 24 cSNPs, all 9 and 10 amino acid peptides that contained the cSNP allele that would encode the predicted *recipient* peptide were tested for possible binding to HLA-A0201 using the IEDB SMM algorithm (www.immuneepitope.org) [26] and the SYFPEITHI (www.syfpeithi.org) [27] algorithm. Any cSNP with an associated peptide that reached our loose binding criteria (IEDB IC_50_<1,000 nM or SYFPEITHI score ≥17) was selected as a potential HLA-A2 epitope and possible mHA. From the 24 cSNPs, we identified 15 that had at least 1 candidate HLA-A2 restricted epitope according to IEDB or SYFPEITHI. From the 15 identified cSNPs, we ordered 23 peptides with 17 subsequently being made at high purity (Table 2). Two of the 23 ordered peptides, P6 and P20 (Table 2), did not meet our loose binding criteria but were selected to further assess the binding algorithms' performance characteristics. Six of the 23 peptides that were ordered could not be synthesized efficiently because of their hydrophobicity and the resulting difficulty with purification.

**Figure 2:**
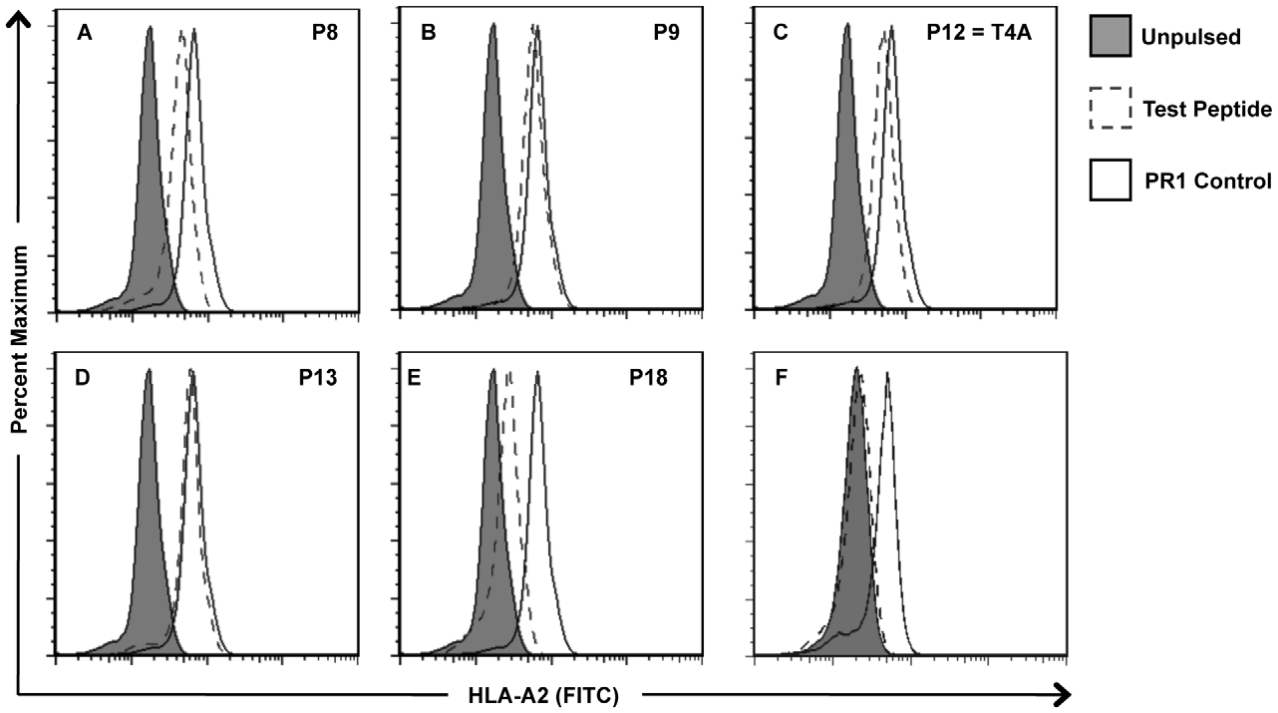
Peptide binding to HLA-A2. All synthesized peptides from Table 2- were tested for binding to HLAA2 using a T2 cell binding assay. Unpulsed T2 cells are shown as shaded traces. Test peptide-pulsed T2 cells are shown as traces with dashed lines, and PR1-pulsed T2 cells are shown as solid black lines. The PR1 peptide was used as a positive control because it binds HLA-A2 with high affinity. Five of 17 peptides bound to HLA-A2 with an increase in geometric mean fluorescence of >1.5 fold compared to the non-pulsed control (2A–2E). A representative non-binding peptide is shown (2F).

Using a T2 cell-based binding assay as a screening test for peptide-HLA binding, we considered any peptides that induced a geometric-mean fluorescence shift >1.5× the unpulsed control to have evidence of HLA-A2 binding *in-vitro*. Five of the 17 tested peptides bound HLA-A0201 according to our assay (Figure 2A– 2E). Figure 2F shows a representative binding assay of one of the peptides that did not bind HLAA0201 according to our assay. In evaluating our *in-silico* prediction analysis we observed that all peptides predicted to have a dissociation constant of <500 nM by the IEDB SMM algorithm bound HLA-A0201 in our assay conditions, whereas the SYPEITHI algorithm was not predictive in our hands (Table 2).

### Evidence of T4A-specific CD8+ T cell expansion in post-SCT samples

**Figure 3:**
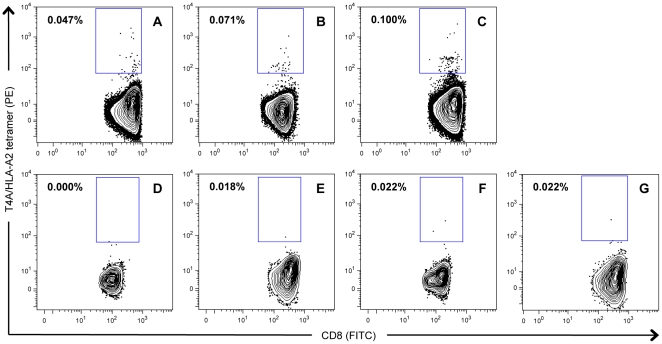
T4A-specific CD8+ T cell expansion in post-SCT patient samples. T4A-specific CD8+ T cells are detected in post-transplant samples using T4A loaded, HLA-A2/PE conjugated tetramers. Of four patients with gIR+ to the cSNP at position rs9876490_C with available post-SCT PBMC samples, T4A-tetramer+ CD8+ lymphocytes were identified in 3 patients (3A–3C). The fourth patient with no detectable T4A-specific T lymphocytes was receiving systemic steroids for extensive cGvHD (3D). No T4A/HLA-A2 tetramer+ cells were detected in a control HLA-A2 negative recipient (3E), or in two HLA-A2+ post-transplant T4A gIR- patients (3F, 3G). The mean percentage of T4A-tetramer+ cells in the CD8+ population in samples 3A, 3B, and 3C was 0.073±0.015% compared to 0.021±0.001% for samples 3E, 3F, 3G (student t-test, P = 0.0278).

To look for evidence of candidate mHA-specific T lymphocyte activity, we first synthesized PE-conjugated/HLA-A0201 tetramers for each of the 5 binding peptides shown in Figure 2. We then performed tetramer analysis on posttransplant PBMC samples that were ≥3 months post-transplant, and were predicted to have a gIR+ to one of the predicted mHA. For the 5 tetramers analyzed we identified between 2 and 5 samples that met our criteria. For peptides 8 and 9 (derived from the same cSNP) we tested 4 gIR+ samples and found no tetramer specific responses. For peptide 13 we tested 2 gIR+ samples and found no tetramer-specific responses, and for peptide 18 we tested 3 gIR+ samples and found no tetramer-specific responses. We identified 5 post-SCT samples that had gIR+ to the 12^th^ peptide synthesized (P12, GLYTYWSAGA); however, 1 of these samples had poor viability and could not be analyzed by flow cytometry. In 3 of the remaining 4 samples examined we identified distinct tetramer+ populations in the live, CD8+, ‘dump’− gate (Figure 3A– C). The sample that did not have a tetramer+ population was obtained from a patient 9 months post-transplant who was on systemic steroids for extensive cGvHD (Figure 3D). We did not identify any tetramer+ populations in an HLA-A2- control (Figure 3E), or 2 HLA-A2+, 3-month post-transplant samples from patients genetically predicted to be gIR- to GLYTYWSAGA (Figure 3F, 3G). The P12 peptide, renamed T4A, is derived from the alanine allele on cSNP rs9876490_C, which is contained in the protein TRIM42.

### T4A binding, tissue expression, and antigen frequency

**Figure 4:**
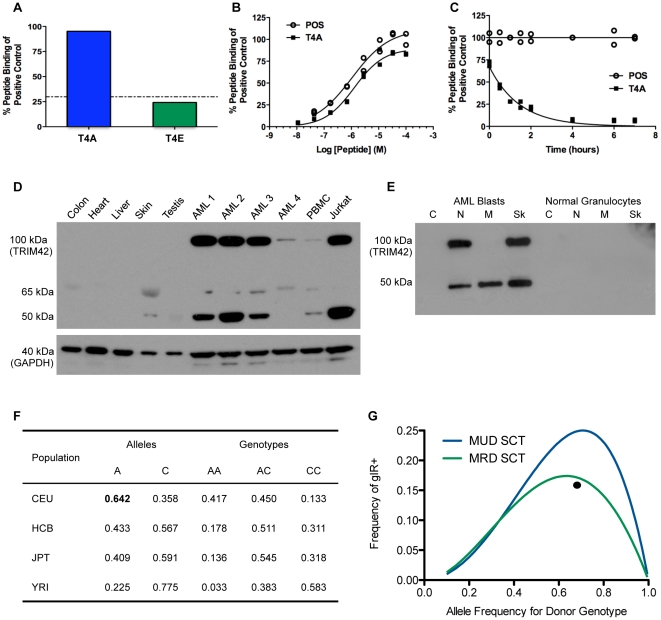
T4A binding, tissue expression, and antigen frequency. The iTopia epitope binding assay was used to measure T4A and the alternately encoded T4E peptide (GLYTYWSAGE) binding to HLA-A2 compared to the percentage of maximal binding of the iTopia control peptide (FLPSDFFPSV). T4A binding to HLA-A0201 is 95% of that compared to the positive control; however, T4E binds HLA-A0201 at 24% that of the positive control. According to the manufacturer, peptides binding of >30% that of the control peptide are candidate HLA-A0201 epitopes (4A). The iTopia assay was also used to measure the ED_50_ and dissociation t_1/2_ of T4A to HLA-A2 relative to the iTopia positive control (FLPSDFFPSV) labeled “POS”. T4A binds with a 50% effective dose of ED_50_ = 1.3 µM and a dissociation half time of t_1/2_ = 1.00 hr (4B, 4C). Western blots on whole cell lysates of normal human tissue (colon, heart, liver, skin, testis and PBMC), 4 human AML samples and Jurkat cells (positive control) are shown (4D). Western blotting of Jurkat cells reveals 2 bands of roughly 100 kDa (full length TRIM42) and 50 kDa (unknown protein product). The TRIM42 band is seen at various levels in all AML samples and faintly in PBMC. No TRIM42 protein is detected in the other human tissues. GAPDH expression (40 kDa) was used as a loading control. Subcellular fractionation was performed on AML blasts and isolated healthy donor granulocytes (4E). The fractions are labeled C, cytosolic; N, nuclear; M, membrane; Sk, cytoskeletal. No TRIM42 protein is observed in granulocytes, but the same bands seen in the Jurkat cell positive control are detected in the N and Sk fractions of the AML blasts. The allele frequencies and genotypes for rs9876490, the T4A associated cSNP, in the original ethnic populations used by the International HapMap Project (CEU, HCB, JPT, YRI) are shown (4F). The T4A recipient phenotypes (and frequencies in the CEU population) are AC (0.450) or CC (0.133), and the donor genotype is AA (0.417). The predicted gIR+ in an unrelated transplant scenario can be calculated: P(AA) × P(AC + CC) and applied over a range of allele frequencies yielding the MUD SCT curve (4G). When parental genotypes are considered in the MRD SCT setting (Supplement 1) a similar curve with generally lower gIR+ frequency at each donor allele frequency is generated (4F). In the case of rs9876490 the T4A gIR+ frequency observed in our cohort (•) fits the predicted MRD SCT curve well.

After T4A-specific responses were observed in post-SCT samples we further characterized T4A's binding properties. The T4A peptide binds HLA-A0201 with high affinity when compared to a positive control peptide; however, the analogous peptide encoded by the alternate allele, rs9876490_A, (T4E  =  GLYTYWSAGE) did not bind HLA-A0201 (Figure 4A). This finding is consistent with a minor antigen mismatch as donors who are homozygous for rs9876490_A and are only capable of producing T4E will be intolerant to T4A because their T lymphocyte repertoire will not have been exposed to the GLYTYWSAG motif because the C-terminal glutamate residue prevents proper binding to HLA-A0201. T4A's ED_50_ is 1.3 µM, and the dissociation half time, t_1/2_, is 1.00 hr (Figure 4B, 4C).

**Figure 5:**
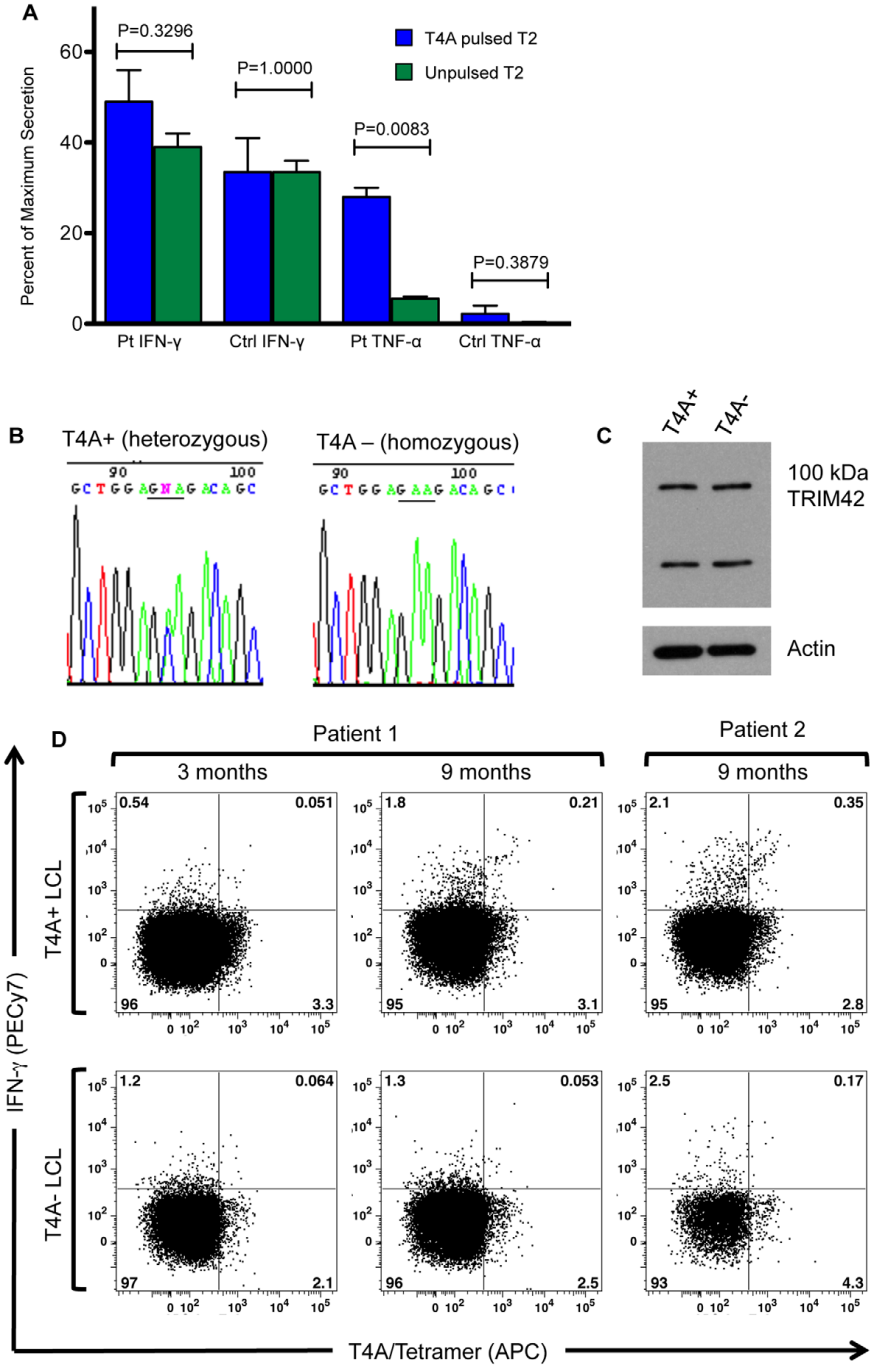
T4A-specific cytokine secretion in post-SCT patient samples. T2 cells were pulsed with T4A peptide and incubated with a rs9876490 heterozygous control and a T4A gIR+ post-SCT sample. IFN-γ and TNF-α secretion were measured by Luminex analysis and normalized to maximum secretion induced by incubation with OKT3. The T4A gIR+ post-SCT sample had a greater TNF-α (P = 0.0083) secretion and a non-statistically greater IFN-γ secretion (student t-test, P = 0.3296) when incubated with pulsed T2 cells vs. non-pulsed T2 cells (5A). This difference was not observed in the healthy control sample (5A). Two HLA-A0201 expressing EBV-LCL lines were generated and genotyped using Sanger sequencing. One EBV-LCL line was heterozygous for rs9876490 and therefore capable of generating T4A (T4A+), and the other was homozygous for rs9876490_A and therefore incapable of generating T4A (T4A−) (5B). Both EBV-LCL lines produce full length TRIM42 (5C). Two T4A gIR+ patients were identified and post-SCT PBMC samples were split and then incubated with both EBV-LCLs. In both patients an increased T4A/tetramer+, IFN-γ+ population was identified in the sample that was incubated with the T4A+ EBV-LCLs and not the T4A- EBV-LCLs (5D).

Because of concerns about immune-mediated toxicity from mHA-specific CTLs towards non-hematopoietic tissues, we evaluated TRIM42 tissue expression. We performed western blots to probe for TRIM42 protein in normal human tissues and AML samples (Figure 4D). Western blotting for TRIM42 in Jurkat cells, a positive control, revealed two bands of roughly 100 kDa and 50 kDa. The high MW band is identified as TRIM42 (Abcam reference western blot performed on Jurkat cells), but the identity of the low MW band is unknown. We probed for TRIM42 in 5 human tissues, colon, heart, skin, liver, testis, and 4 human AML samples (Figure 4D). We observed varying amounts of TRIM42 protein in all 4 AML samples, but were not able to detect appreciable amounts of TRIM42 in our human tissues, which included the 3 main target organs for GvHD, colon, skin and liver. We subsequently created subcellular fractions of AML blasts and normal granulocytes to determine the localization of TRIM42 in AML. We could not identify any TRIM42 specific bands in the normal granulocyte subcellular fractions. However, we did observe the same 2 bands identified in our previous western blots in the nuclear and cytoskeletal fractions of the AML sample (Figure 5E). The significance of the low molecular weight band being found without the high molecular weight band in the AML membrane fraction is not known. Some TRIM proteins, including PML/TRIM19, are involved in transcriptional regulation [28], [29], while others such as TRIM25 are involved in innate immunity and anti-viral responses [30], [31], [32]. The function of TRIM42 is not known.

For mHAs to be of clinical utility in a SCT population they must occur at a frequency that is high enough to justify their development as a therapeutic. The TRIM42 cSNP, rs9876490, identified in our study has been annotated through the Human HapMap Project [22], and its allele frequencies for European (CEU), Han Chinese (HCB), Japanese (JPT), and Yoruban, Nigerian (YRI) populations are given in Figure 4F. The population in our study was 90% European in ancestry (CEU). The T4A peptide is encoded by the *recipient* allele, rs9876490_C, and in the CEU population, it is present at a frequency of 0.358. The gIR donor genotype, AA, is present in 41.7% with the rs9876490_A allele being present at a frequency of 0.642. Using Hardy-Weinberg equilibrium, the frequencies of the gIR donor (AA) and gIR recipient (AC, CC) genotypes can be calculated. We generated gIR+ frequency curves for a range of donor allele frequencies in both an unrelated and related SCT (Figure S1, and Figure 4G). These curves show the maximum frequency of gIR+ in an *unrelated donor setting* being 0.25 if the donor genotype (e.g. AA) exists at frequency 0.5 and the recipient genotype (AC, CC) exist at a combined frequency of 0.5. This situation occurs with a donor allele frequency (e.g. A) ≈ 0.7. From the rs9876490 genotype frequencies given in Figure 4F the predicted gIR in an *unrelated*( transplant is 0.243, which is close to the maximum frequency predicted. The analysis for a matched related donor transplant is complicated by the fact that not all parental genotypes e.g. father is CC genotype and mother is AA genotype) can yield sibling offspring with the possibility of having both donor and recipient genotypes (Figure S1). Taking these factors into account, the MRD curve in Figure 4G+ is generated. The maximum gIR frequency is predicted to occur at a donor allele frequency of 0.632 and yield a gIR+ frequency of 0.174. The frequency of a T4A gIR+ in our patient cohort is 0.158 (plotted as the filled circle on Figure 4G) in good agreement with our calculations.

### Evidence of T4A-specific cytokine secretion in post-SCT PBMC

Additional cells from the patient characterized in Figure 3C were available so we tested T4A's ability to induce cytokine secretion in this patient and in a rs9876490 heterozygous healthy control. After incubating patient and healthy donor PBMC with T4A-pulsed T2 cells we measured IFN-γ and TNF-α secretion using Luminex analysis. Cytokine secretion is normalized to maximal cytokine secretion elicited by OKT3 antibody (Figure 5A). This patient sample had a T4A specific TNF-α response primarily, and there was a non-statistically significant increase in IFN-γ secretion in response to T4A also. The relatively modest cytokine responses may in part be a reflection of the small number of T4A-specific CD8+ cells identified in this patient. IFN-γ and TNF-α secretion were not significantly higher in response to T4A in the healthy donor.

Because the above assay did not evaluate functional responses to a target cell that could endogenously produce T4A we performed tetramer-cytokine flow cytometry on two additional post-SCT patients. We first generated HLA-A0201 expressing, Epstein-Barr virus immortalized, lymphoblastic cell lines (EBV-LCLs) that would serve as the source of endogenous T4A peptides. After the lines were established we genotyped each line for the rs9876490 cSNP. We identified one EBV-LCL line as heterozygous for rs9876490 so it could theoretically present the T4A peptide, and another EBV-LCL line was homozygous for rs9876490_A (i.e. the donor genotype) so it could not present T4A (Figure 5B). We then confirmed that both cell lines produced equivalent amounts of TRIM42 protein by western blot (Figure 5C). We identified 5 frozen PBMC samples that had been obtained ≥3 months post-SCT from 4 patients, not receiving systemic steroids at the time of PBMC collection (2 from 1 patient and 1 each from the other 3 patients), who were T4A gIR+. Each sample was split with ½ being incubated with T4A+ EBV-LCL and the other ½ incubated with T4A- EBV-LCL. After incubation, we performed tetramer-cytokine flow cytometry on the samples. Two samples were too lymphopenic to evaluate, but the 3 evaluable samples are shown (Figure 5D). In both evaluable patients we observed an increased number of tetramer+, IFN-γ+ CD8+ T cells in the 9 months post-SCT samples that had been incubated with the T4A+ EBV-LCLs.

### T4A mismatch and clinical outcomes

**Figure 6:**
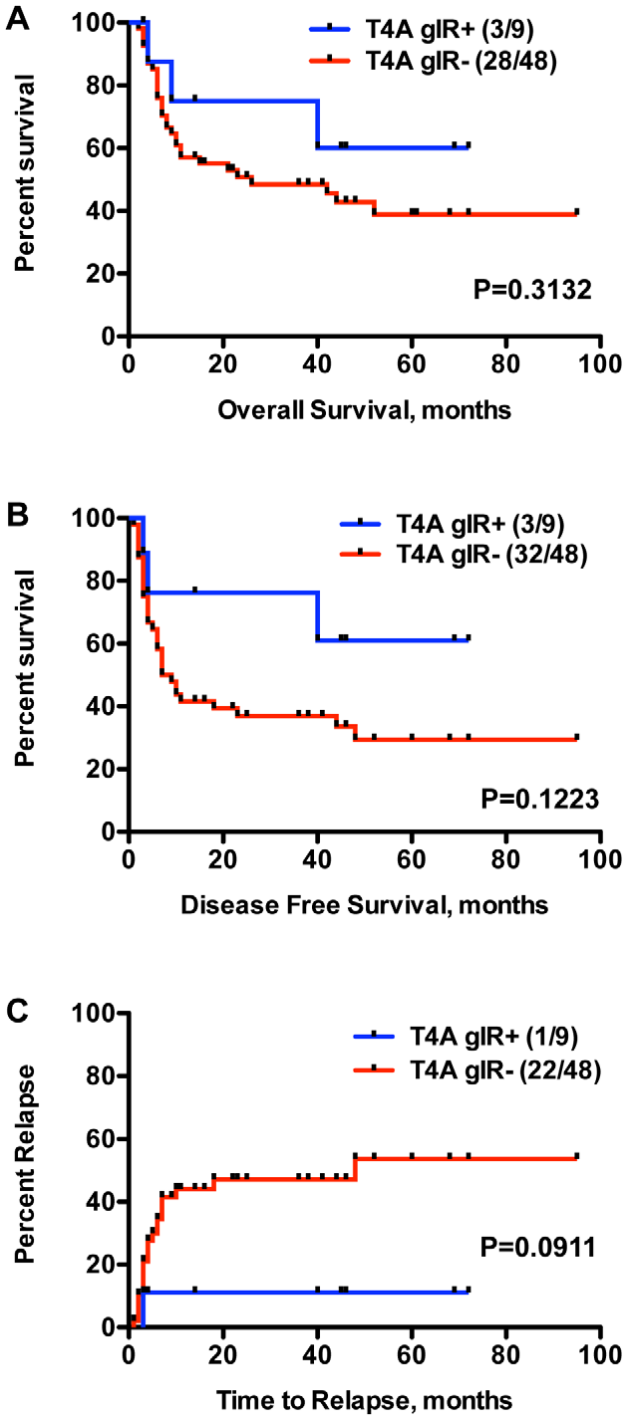
T4A gIR+ and clinical outcomes. A Kaplan-Meier survival analysis for the effect of T4A gIR on OS and DFS was performed on the 57 examined patients. A trend towards improved OS (median OS: NR vs. 26 mos, P = 0.3132) and DFS (median DFS: NR vs. 8 mos, P = 0.1223) was observed in the T4A gIR+ patients compared to T4A gIR- patients (6A, 6B). Also, a trend towards improved relapse rates was observed in the T4A gIR+ patients (11% vs. 46%, P = 0.0911).

Previous investigations have assessed specific mHA mismatches in effecting differences in survival [3], [19], [20]. Because our discovery method was intended to identify high prevalence candidate minor antigens, primarily dependent upon gIR frequency, that are associated with a clinical outcome, in this pilot study cIR, we tested our cohort to see if T4A gIR+ SCT vs. T4A gIR- SCT were associated with survival or relapse differences. All 9 patients in our cohort who were identified as T4A gIR+ had the T4A gIR+ mismatch in the GvL/GvHD direction (i.e. donor homozygous and recipient heterozygous), not the “intolerant” (i.e. donor homozygous and recipient homozygous for the other allele) direction so our analysis was able to evaluate T4A gIR+ in the GvL/GvHD direction without confounding from possible T4A reactions in the rejection direction. In our cohort we observed improved overall (median OS: Not Reached, NR, vs. 26 mos, P = 0.3132) and disease free (median DFS: NR vs. 8 mos, P = 0.1223) survival in the T4A gIR+ cohort; however, neither outcome met statistical significance primarily because of our limited sample size. (Figure 6A, 6B). We also observed a non-statistically significant trend towards improved relapse rate (Figure 6C) in the T4A+ vs. T4A- cohorts (11% vs. 46%, P = 0.0911). There were no statistically significant differences in the T4A gIR+ and T4A gIR- cohorts with respect to age, disease, disease stage, frequency of gender-mismatch transplant, and CMV incidence (data not shown). Five of the 9 T4A gIR+ patients did not have ≥ grade 2 aGvHD or extensive cGvHD, suggesting that T4A gIR+ is unlikely to be a strong independent mediator of GvHD.

## Discussion

The importance of mHAs in mediating GvL and GvHD in the allo-HSCT setting has been known for roughly 20 years [2], [4]. Traditional T cell cloning, used to identify these potentially important immune targets have yielded relatively few mHA over this time frame. One of the main limitations of this approach is that there is no way to characterize the properties of the mHA until after the T cell is cloned. As a result, significant resources can be used generating a T cell clone and characterizing its cognate mHA that, eventually, may not be a good candidate for therapeutic considerations [5], [12], [13], [18]. One possible method to address this limitation is to use genomics and bioinformatics filtering strategies to prioritize candidate mHA for discovery. This paper describes one approach to this strategy: combining genetic prevalence data with clinical outcomes and bioinformatics to rank possible mHA with subsequent confirmation in patient samples.

Our screen is primarily based upon prioritizing candidate mHA based upon their predicted frequency in a population. This preliminary ranking is based upon gIR, and candidate mHA could theoretically be ranked based purely upon donor allele frequencies and their resultant gIR (see Figure 4G), Figure S1. Using gIR as the sole criteria for cSNP ranking would have yielded 6,102 cSNPs having gIR+ ≥0.2 and 2,301 cSNPs having gIR+ ≥0.243 – equal to T4A's predicted gIR. This number of cSNPs and possible mHA is prohibitively large for detailed characterization so to further refine our mHA selection we used Fisher's exact test to associate a clinical outcome, cIR, with the measured gIR in out patient cohort (Figure 1). This approach enabled us to simplify our search for common mHA that were most closely associated with a clinical outcome. By using this approach with subsequent bioinformatics filtering we were able to identify a high prevalence mHA in 3 post-SCT patients using very small numbers of reagents – 23 ordered peptides and 5 synthesized tetramers.

Because our method theoretically screened 528,846 (13,917×2 alleles ×19 possible decameric and nonameric peptides containing the allele) candidate HLA-A2 restricted epitopes represented by 13,917 cSNPs on the Illumina NS-12 array, this analysis does encounter the problem of multiple testing. If we were to test 13,917 independent cSNPs for a specific outcome (e.g. cIR), the most conservative test for statistical significance would be to divide our pre-defined threshold for statistical significance (e.g. P<0.05) by 13,917, which would yield a P-value ≈ 3×10^−6^. Our method cannot achieve this level of statistical power for several reasons. In addition to our limited cohort size, which will obviously hinder statistical power, our method of using Fisher's exact test to measure the association between gIR and cIR yields relatively high P-values because in the matched related setting there are only predicted to be, at most, 17% gIR+ donor/recipient pairs. More importantly, clinical outcomes, including cIR status, are dependent upon many well-established clinical variables, such as age, disease and its associated prognostic factors (e.g. cytogenetics), and disease status at transplant. Under the most optimistic circumstance, where no gIR+ donor/recipient pair have a clinical outcome of cIR-, we estimate a sample size of roughly 300 being needed to achieve P<3×10^−6^, and given the many other clinical risk factors for cIR- outcomes, to fully assess the association between a cSNP encoded mHA and cIR would require significantly more samples than we have available at this time. Because of these sample size considerations, we used our combined clinical and genomic ranking data with a bioinformatic and an *in-vitro* testing method to refine our list of candidate peptides to a manageable number, 40, for testing in post-SCT patient samples.

While our study purports to use both a statistical analysis of genomic and clinical outcomes with refinement through bioinformatics methods to discover common clinically useful mHA, the relative utility of gIR, cIR and bioinformatics processing are not entirely clear. gIR data simply allow ranking by probability of genetic minor antigen mismatches, and cIR data are susceptible to multiple clinical covariates and sample size limitations. To investigate the utility of using both gIR and cIR data (Figure 1C) to initially prioritize cSNPs, we performed simulations where donor/recipient pairs were randomly moved between cIR outcome groups. We performed 3 such simulations and observed that our original clinical data possessed the most cSNPs with low P-values (i.e.<0.0375) compared to any of the other random simulations (Figure S2). The result suggests that our ranking of cSNPs based upon association between gIR and cIR is better than random chance and supports our view that combining cIR data to microarray based gIR results is unlikely to reduce mHA predictive power and can help in the initial prioritizing of candidate mHA that should be taken on for further testing. The fact that our simple bioinformatics approach eliminated 16 of the original candidate cSNPs before any testing, and the IEDB algorithm successfully predicted the 5 HLA-A0201 binding peptides and rejected the other 18 ordered peptides, point to the power that the follow-up bioinformatics approach had in reducing the number of peptides that need to be ordered and tested in these type of antigen discovery projects.

T4A is a high gIR+ frequency (predicted MUD gIR+  = 0.243, measured MRD gIR+  = 0.158)) mHA that strongly associated with our defined clinical outcome, cIR, which focuses on immune-mediated outcomes such as remission, relapse, and GvHD, our primary interest, and minimizes the impact of non-immune specific outcomes such as treatment related mortality from conditioning regimens and infectious complications which could have affected other clinical metrics such as OS and DFS. Our T4A survival analysis provides evidence that the cIR composite outcome reflects some information regarding OS, DFS, and relapse; however, our method can be easily modified to test cSNP gIR correlations with other clinical outcomes such as long-term remission without GvHD, development of GvHD or even OS.

While the T4A gIR+ frequencies are close to the maximum predicted for a cSNP, our patient sample size prevents us from drawing definitive conclusions about T4A gIR+ effect on survival and relapse outcomes. A power calculation for a two-sided test with a type 1 error rate of 0.05 and a power of 80% to detect a median survival improvement of 50% would require the T4A gIR+ cohort to have 96 subjects. Because T4A gIR+ should occur in 17% of our MRD patients we would need a total of 565 subjects (96 predicted to be T4A gIR+ and 469 predicted to be T4A gIR-), numbers that are not available except through cooperative groups.

Our study did not identify several of the previously well-characterized mHAs; however, this is likely because we used an off-the-shelf microarray and a ranking method that penalizes cSNPs with low allele frequencies. Both the HA-1 and HA-2 cSNPs are not included in the Illumina NS-12 chip so we cannot evaluate them in this analysis. One illustrative example however is the mHA LB-ADIR-1F. This mHA is associated with the cSNP rs2296377. The cSNP has high minor allele, T, frequency (MAF = 0.213), and this allele is the mHA encoding the recipient peptide. Consequently the C allele is the donor allele, and it has an allele frequency of 0.787 [12], [14]. This distribution is close to the ideal distribution of having a donor allele frequency of 0.7. As expected, we observed many, i.e. 11, LB-ADIR-1F gIR+ in the 57 patient test cohort; however, 2 of the 11 LB-ADIR-1F gIR+ were identified in the cIR- cohort. This distribution gave a P-value of P = 0.705. If all 11 LB-ADIR-1F gIR were associated with the cIR cohort the P-value would have been 0.048 similar to the P-value for T4A, which had all gIR+ associated with cIR+. These dramatic changes are a result of our relatively small sample size and would be mitigated if a larger cohort of patients were examined.

This study demonstrates how using genomics data combined with clinical outcomes and bioinformatics pipelines can guide mHA discovery so that common mHAs with desirable clinical properties can be preferentially selected for further evaluation as possible prognostic [3], [19], [20] or therapeutic targets [5]. Each individual test that we employed can be readily optimized. Microarray analysis is becoming less expensive, and through genome imputation methods [33] data for all of the high frequency cSNPs annotated through the 1000 Genomes Project can be encompassed. Clinical data and outcomes will be more accurate as larger SCT populations are followed for longer periods of times. Finally, bioinformatics will allow increasingly accurate epitope prediction algorithms [34] to more accurately predict epitopes for testing and also enable further filtering of candidate cSNPs according to features such as tissue expression.

## Supporting Information

Figure S1 Frequencies of Genetic Predictors of Minor Histocompatibility Mismatches.
(PDF)

Figure S2 Clinical outcome data yields more cSNPs strongly associated with gIR than predicted by random association.
(PDF)
