# Supplementary material for: Common Minor Histocompatibility Antigen Discovery Based upon Patient Clinical Outcomes and Genomic Data
Source: PLoS One. 2011 Aug 9;6(8):e23217. doi: 10.1371/journal.pone.0023217 (PMC3153501; doi:10.1371/journal.pone.0023217)
Supplement: Figure S1 — Frequencies of Genetic Predictors of Minor Histocompatibility Mismatches. (PDF) [file pone.0023217.s001.pdf]

Figure S1

Matched Unrelated Donor Transplant:

In an unrelated donor transplant we will assume that alleles will distribute according to Hardy-Weinberg equilibrium at the frequency for the ethnic groups being used. The possible donor and recipient combinations for an unrelated donor transplant are:

D \ R	**AA** p^2	**Aa** 2pq	**aa** q^2
**AA** p^2	Tolerant (p^4)	GvL/ GvHD ($2p^3q$)	Non-tolerant (p^2q^2)
**Aa** 2pq	Rejection ($2p^3q$)	Tolerant ($4p^2q^2$)	Rejection ($2pq^3$)
**aa** q^2	Non-tolerant (p^2q^2)	GvL/ GvHD ($2pq^3$)	Tolerant (q^4)

Genetic predictors of an immune response (gIR) occur when a homozygous donor (AA) with allele frequency p^2 is transplanted into a genetically non-identical recipient (Aa, or aa) with allele frequencies of 2pq (for Aa) or q^2 (for aa). For a given cSNP with a donor allele, A, with donor allele frequency, p, we can define a "recipient" allele (i.e. the allele the encodes the mHAg peptide), a, to have a frequency, $q = 1 - p$.

The frequency of gIR can therefore be determined as the product of the frequencies of the donor genotype (AA) and the frequencies of the summed "recipient" genotypes (Aa, aa) as follows.

$$gIR = p^2 \times (2pq + q^2)$$

This yields the following curve:

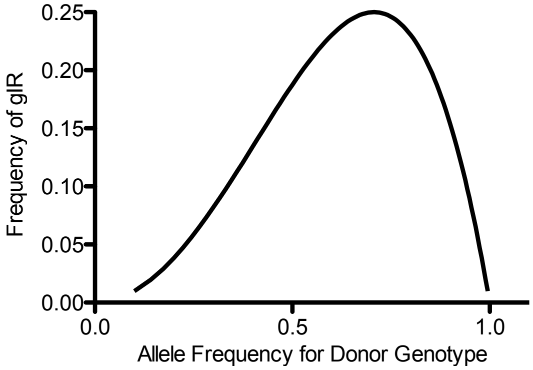

This curve shows that the donor allele frequency (A) that would be expected to yield the maximum frequency of gIR in an unrelated transplant setting is ≈ 0.7 because this yields a donor genotype (AA) frequency of ≈ 0.49. Therefore, the recipient genotypes (Aa, and aa) are expected to exist at a frequency of ≈ 0.51. This product of frequencies yields a theoretical maximum frequency of gIR of 0.25.

Figure S1

Matched Related Donor Transplant:

In a matched related donor transplant calculation of the frequencies is complicated by the fact that only certain parental genotypes can yield offspring where a sibling pair can have a homozygous donor (AA) and a recipient genotype (Aa, aa). The possible parental genotype combinations that can yield gIR between sibling offspring are AA×Aa and Aa×Aa.

The probability for a maternal (M) and paternal (P) genotype pair that can yield both donor and recipient genotype offspring can be written as:

$$P(\text{parental pair}) = 2\times[P(AA\times Aa) + P(Aa\times Aa)] = 2\times[(p^2\times 2pq) + (2pq\times 2pq)]$$

The probabilities are multiplied by 2 to account for the fact that combinations can occur with either parent having the donor genotype.

The probability of a sibling pair creating a gIR depends upon the parental genotypes. For the parental pair who are AA and Aa the probability of there being a sibling pair with one donor genotype and one recipient genotype is 0.25 with 0.5 probability of donor genotype (AA) and 0.5 probability of recipient genotype (Aa).

For the parental pairs who are both Aa and Aa the probability of there being a sibling pair with one donor genotype is 0.1875 with 0.25 probability of donor genotype (AA) and 0.75 probability of recipient genotype (Aa, aa).

$$P(gIR) = 2\times[0.25\times(p2\times 2pq) + 0.1875\times(2pq\times 2pq)]$$

This equation yields the following curve:

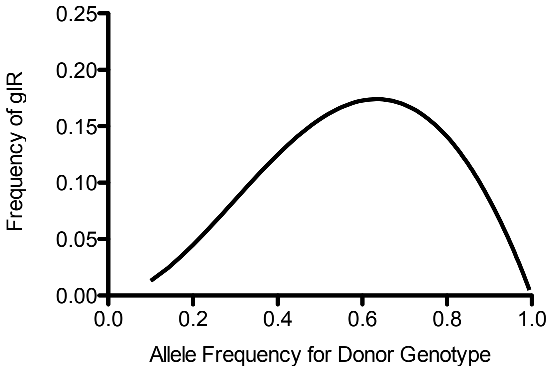

Similar to the case of the matched unrelated stem cell transplant the maximum frequency of gIR occurs with a donor allele frequency close to 0.7 (0.63); however, the maximum theoretical gIR frequency is only 0.174, which is the gIR frequency of T4A in our study.
