# Supplementary material for: Common Minor Histocompatibility Antigen Discovery Based upon Patient Clinical Outcomes and Genomic Data
Source: PLoS One. 2011 Aug 9;6(8):e23217. doi: 10.1371/journal.pone.0023217 (PMC3153501; doi:10.1371/journal.pone.0023217)
Supplement: Figure S2 — Clinical outcome data yields more cSNPs strongly associated with gIR than predicted by random association. (PDF) [file pone.0023217.s002.pdf]

Figure S2

To investigate the role the clinical outcomes played in helping rank cSNPs in this study we performed randomization simulation to study the number of cSNPs that yielded a high association between gIR and clinical outcomes (cIR+ and cIR-). We first determined the actual P-values for the top 25 ranked cSNPs using the clinical data we had to group patients into the cIR+ and cIR- cohorts. We then performed 3 separate randomization procedures were each donor / recipient pair was randomly assigned to either the cIR+ and cIR- cohorts. The randomization was created so that the number of pairs in the cIR+ and cIR- cohorts was the same in the 3 randomization simulations as the original clinical data.

The results of the original analysis and the 3 simulations are shown in the graph below:

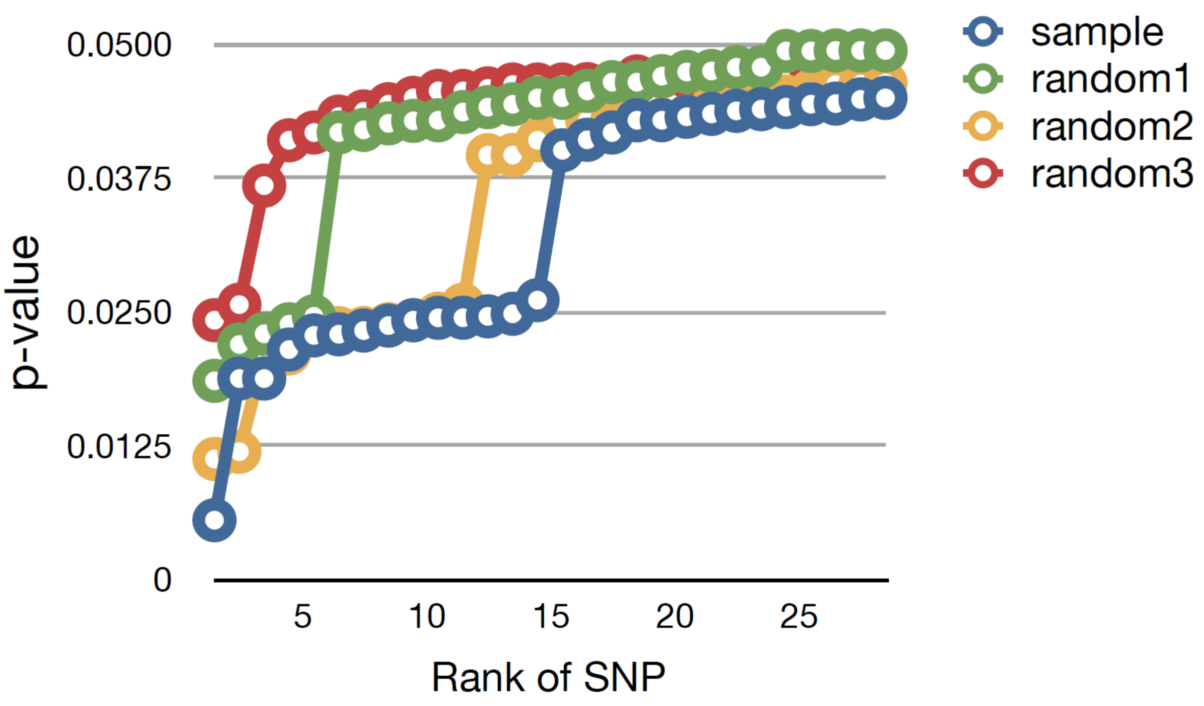

The curves show that the original data (sample), the top 13 cSNPs have P-values < 0.025, while the 3 simulations all have fewer cSNPs with this low P-value.

This analysis suggests that our method gains some additional selection power based upon the clinical ourcomes data that we used; however, to fully address this question a much larger cohort of patients would be needed.
